# Advances in Nuclear Radiation Sensing: Enabling 3-D Gamma-Ray Vision

**DOI:** 10.3390/s19112541

**Published:** 2019-06-04

**Authors:** Kai Vetter, Ross Barnowski, Joshua W. Cates, Andrew Haefner, Tenzing H.Y. Joshi, Ryan Pavlovsky, Brian J. Quiter

**Affiliations:** 1 Department of Nuclear Engineering, University of California, Berkeley, CA 94720, USA; rossbar15@gmail.com; 2 Applied Nuclear Physics, Lawrence Berkeley National Laboratory, Berkeley, CA 94720, USA; JCates@lbl.gov (J.W.C.); ahaefner@lbl.gov (A.H.); thjoshi@lbl.gov (T.H.Y.J.); RTPavlovsky@lbl.gov (R.P.); bjquiter@lbl.gov (B.J.Q.)

**Keywords:** nuclear radiation mapping, 3-D nuclear sensing, gamma-ray imaging, gamma camera, gamma-ray vision, scene data fusion, unmanned systems, range-finding, SLAM

## Abstract

The enormous advances in sensing and data processing technologies in combination with recent developments in nuclear radiation detection and imaging enable unprecedented and “smarter” ways to detect, map, and visualize nuclear radiation. The recently developed concept of three-dimensional (3-D) Scene-data fusion allows us now to “see” nuclear radiation in three dimensions, in real time, and specific to radionuclides. It is based on a multi-sensor instrument that is able to map a local scene and to fuse the scene data with nuclear radiation data in 3-D while the instrument is freely moving through the scene. This new concept is agnostic of the deployment platform and the specific radiation detection or imaging modality. We have demonstrated this 3-D Scene-data fusion concept in a range of configurations in locations, such as the Fukushima Prefecture in Japan or Chernobyl in Ukraine on unmanned and manned aerial and ground-based platforms. It provides new means in the detection, mapping, and visualization of radiological and nuclear materials relevant for the safe and secure operation of nuclear and radiological facilities or in the response to accidental or intentional releases of radioactive materials where a timely, accurate, and effective assessment is critical. In addition, the ability to visualize nuclear radiation in 3-D and in real time provides new means in the communication with public and facilitates to overcome one of the major public concerns of not being able to “see” nuclear radiation.

## 1. Introduction

Three-dimensional (3-D) gamma-ray and X-ray vision has been mainly the matter of science fiction and comic books in the past. However, recent advances in computer vision along developments of compact nuclear radiation detectors and imagers enable the reconstruction of scenes fused with nuclear radiation in 3-D, providing new means to visualize gamma radiation and to enable gamma-ray vision in 3-D. Computer vision is at the core of numerous technologies to automate, analyze, learn, or control processes or features; nuclear detection and imaging is at the core for the detection and mapping of radioactive materials, relevant for applications ranging from medicine and physics to nuclear security and safety. The combination of both complementary technologies enables unprecedented and “smarter” ways to detect, map, and visualize nuclear radiation fields. Scene-data fusion (SDF) represents the realization of this combination, by integrating “contextual” sensors, such as visual cameras or light detection and ranging (LiDAR), and nuclear radiation detection and imaging instruments [1,2,3,4,5,6]. It enables the mapping and visualization of gamma radiation in 3-D in the context of local scenes while the instrument is being moved through this scene. Since we are able to measure the energies of the gamma-rays, maps of specific radionuclides can be created. This is due to the fact that gamma-ray energies serve as fingerprints of specific radionuclides. For example, maps of cesium, cobalt, or iodine isotopes can be created simultaneously, which is important to guide the response to intentional or un-intentional releases of radioactive materials.

Fusing complementary imaging modalities is not new. For example, in biomedical imaging, X-ray imaging—revealing anatomical features—has been combined with radionuclide imaging—revealing functional or physiological features. The fusion of both modalities provides unparalleled means to diagnose disease, to monitor therapy, and to develop pharmaceuticals to cure a wide range of diseases [7,8,9,10,11]. A static gantry is sufficient as the subject of study can be brought to the instrument. In astrophysics, or more specifically, in gamma-ray astronomy, gamma-ray telescopes are used to study the evolution of matter and stars in a pseudo-static fashion, as the instrument and the object of study do not move quickly relative to each other. Large scale, complex, and expensive gamma-ray telescopes have been developed and successfully launched in space or on high-altitude balloons over the last 30 years, providing a look into the extra-terrestrial gamma-ray world at energies ranging from about 100 keV up to several GeV [12,13,14,15,16,17,18]. Ground-based, so-called Imaging Atmospheric Cherenkov Telescopes image gamma-rays through the observation of Cherenkov radiation, which originate from interactions of gamma-rays up to 50 TeV in the atmosphere [19,20]. The gamma-ray image is often overlaid with images obtained in wavelengths ranging from microwave to X-ray (or from cm to pm in terms of wavelength). It is worth noting that in these gamma-ray telescopes, the gamma-ray energy is measured providing a fingerprint of specific radionuclides, the shape of the spectral continuum, or the amount of annihilation radiation associated with physics processes.

In contrast to gamma-ray astronomy, in many circumstances related to the search and mapping of radiological or nuclear materials in terrestrial environments, the mobile deployment of radiation detection and imaging systems are required. One sometimes distinguishes between nuclear and radiological materials, with nuclear materials referring to heavy radioactive isotopes or radioisotopes that are associated with nuclear power or nuclear weapons, such as U-235, U-238, or Pu-239. Radiological materials refer to all other radioisotopes, including the ones used in medicine, such as Tc-99 or I-131 or in other industrial applications such as Cs-137 or Co-60. Over the last 10 years, compact and hand-portable gamma-ray imagers or gamma-ray cameras have been developed to enable gamma-ray imaging and, similarly to the space-based gamma-ray telescopes, can create two-dimensional (2-D) maps of radioactive materials. These state-of-the art instruments are deployed in a static fashion and are complemented with a visual camera to enable the overlay of the 2-D gamma-ray with a visual image and to correlate the location of radionuclides with specific objects. While these systems are now being integrated into the operation of nuclear facilities, such as nuclear power plants or storage facilities, to detect leaks and radioactive contamination [21,22,23], the static deployment limits its usefulness. SDF overcomes the restrictions of static deployment. Similar to how X-rays are used in biomedical imaging to provide anatomical information, in SDF, contextual sensors are used to create the scene or the anatomy of the environment, which is then fused with the radiological gamma-ray emission information. In contrast to the stationary imaging systems in the clinic, where the X-ray and gamma-ray imaging system move on well-described paths around the object in a gantry, SDF can be realized on a freely moving system, as it automatically tracks its position and orientation in the scene in 3-D. It facilitates the mobile deployment of radiation detection and imaging systems to cover much larger areas, as compared with the static deployment. Furthermore, it enables the 3-D mapping of radiation fields that are fused with contextual scene data with high accuracy while providing real-time feedback to the user and the visualization of the radiation fields in 3-D. It can be deployed on remote and unmanned aerial or ground based platforms, preventing the exposure of operators to risks associated with radioactivity or the operation. This capability and its associated properties are relevant for a wide range of applications in nuclear security and safety, as well as in environmental and legacy management, nuclear safeguards, consequence management, or in the decommissioning of nuclear facilities and nuclear power plants.

In the following sections, we will introduce the basic concept of SDF, followed by several examples to illustrate its capabilities in the search and localization of radiological materials, as well as in the mapping of contamination or the verification of decontamination activities. 

## 2. Materials and Methods

Most radiation mapping systems in use today consist either of non-imaging radiation detection instruments equipped with a Global Positioning System (GPS) or of a radiation imaging instrument that is deployed statically, i.e., not moving while operated. The former method is limited because it only measures the radiation levels at or on the detector itself, the latter is limited because of the lengthy set up and extended measurement times and limited range that can be probed as a result. These mapping systems also only produce 2-D images or 2-D radiation projections. Still today, contamination mapping is mostly done with a dosimeter requiring the operator to stay at one location in order to obtain a reliable dose-rate reading for this location. This reading is then noted on a tablet or sheet of paper which indicates the location as well, often related to a sketch of the local surroundings. We have developed the SDF concept, which overcomes several limitations of this approach. It enables the mapping of gamma-ray emissions and ultimately dose-rates from a moving platform without requiring the lengthy setup needed to co-register (or sync the location of) the instrument and the scene. This results in faster and more accurate measurements of 3-D environments with real-time observations and almost instantaneous feedback. 

**Figure 1:**
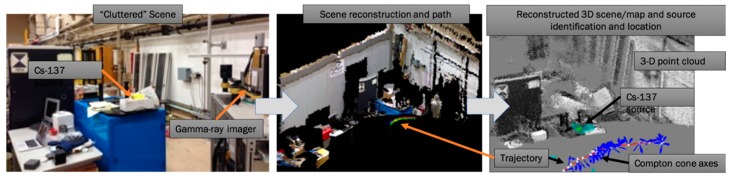
Steps in the creation of fused three-dimensional (3-D) scenes, from the actual cluttered scene (**left**), to the mapped 3-D scene model with the reconstructed path of the instrument (**middle**), and the 3-D scene fused with the reconstructed radiation data (**right**). For this demonstration a high-purity Ge (HPGe)-based gamma-ray imaging instrument mounted on a cart equipped with the Microsoft (MS)-Kinect was used. List-mode maximum-likelihood expectation-maximization image reconstruction was used with the reconstruction constrained to the point cloud created by the structured light sensors.

The methodology of SDF is based on a set of range-finding sensors, radiation detection, and imaging instruments, as well as algorithms for mapping, pose estimation, radiation (image) reconstruction, and data fusion. Range-finding concepts and sensors, such as structured light, light detection and ranging (LiDAR), or visual photogrammetry provide point clouds (or surfaces), reflecting the relative distances between the points (or surfaces) and the instrument, resulting in a 3-D map of the local environment or scene around the instrument [24,25]. For real-time mapping, we used LiDAR with 16 or 32 rotating laser beams which rotate at tens of Hz and produce typically hundreds of thousands of points per second with a range of 100 m and ~cm accuracy [26]. LiDAR measures distances by measuring the time-of-flight of an emitted laser pulse reflected from a surface. Each return pulse creates a point in the scene whose 3-D coordinate is determined by two coordinates of the laser emission and the distance measured. Simultaneous localization and mapping (SLAM) algorithms were used to map and estimate the position and orientation—the pose—of the instrument within milliseconds [27]. A specific SLAM implementation being utilized is the robotics operations system integration of Google Cartographer [28]. The combination of range-finding sensors and SLAM is widely being used to control and navigate autonomous systems. In order to make the mapping and tracking of the platform with SLAM more robust, we complement the LiDAR with GPS and inertial measurement units or IMUs. Knowing the pose of the radiation detection or imaging instrument in the scene or map that is being created allows the backprojection of the counts or image information into the scene. This can be done while the system is being moved through the scene. Figure 1 illustrates the data processing steps employed to produce the 3-D image of the scene, to estimate the position and orientation of the instrument, and to reconstruct gamma-ray events and fuse the complementary data to represent the fused 3-D scene. In this scenario, we are using a gamma-ray imaging system in combination with a MS-Kinect with both mounted on a mobile cart (left panel). The gamma-ray imager consists of two high-purity Ge (HPGe) detectors in double-sided strip configuration, providing 3-D position resolution of <1 mm^3^ for individual gamma-ray interactions [29]. Each detector is characterized by a dimension of 76 × 76 × 15 mm^3^ and by an energy resolution of about 0.2% at 662 keV. The structured light sensor of the MS-Kinect provides the point cloud that is colored by the colors obtained from the visual camera [30]. The colorized point cloud represents the 3-D scene that is created while moving the cart through the scene. At the same time, the SLAM algorithm provides the pose, and with that the trajectory of the cart (middle panel). The right panel shows the reconstructed 3-D scene, now with the color contour representing the reconstructed gamma-ray emission intensity which is embedded into the point cloud. The color contours correctly point to the Cs-137 source. The red line represents the reconstructed trajectory; the white circles show locations that are associated with gamma-rays used for the gamma-ray reconstruction; and finally, the blue arrows indicate the direction of observed gamma-ray scattering in the detector, which is used to reconstruct the angles of incident gamma-rays required for the image reconstruction. In this example, we used the so-called Compton imaging concept which allows us to reconstruct the angles of incident gamma-rays without a collimator (e.g., [31,32]). List-mode maximum-likelihood expectation-maximization (LM-ML-EM) image reconstruction was used in the gamma-ray image reconstruction. Maximum-likelihood expectation-maximization (ML-EM) algorithms are widely used in image reconstruction with underlying Poisson statistics [33]. LM-ML-EM is used when the dimensionality of the measurements makes the reconstruction intractable, as can be found here, when Compton imaging is used on a freely moving system [34]. The activity of the source was 50 μCi, the length of the trajectory was about 5 m, and the measurement time was about 2 minutes. During this time, 94 gamma-ray events were reconstructed to localize the source in 3-D. The ability to localize the source with only 94 events is driven by two properties: (1) The excellent signal-to-background ratio in the energy window that was used (at 662 keV, reflecting the gamma-ray energy of Cs-137); (2) The image reconstruction only into voxels that are occupied by points in the point cloud. The latter significantly improves the contrast, accuracy, and speed in the image reconstruction. It is worth mentioning that the reconstruction does not have to be constrained to specific points, pixels, or voxels and can therefore reconstruct source locations inside or outside of objects. Constraining the reconstruction to only occupied voxels assumes the gamma-ray emission to originate only from within or from the surface of objects that are mapped. This assumption is justified in most scenarios when the radiological material is not dispersed in the air. 

**Figure 2:**
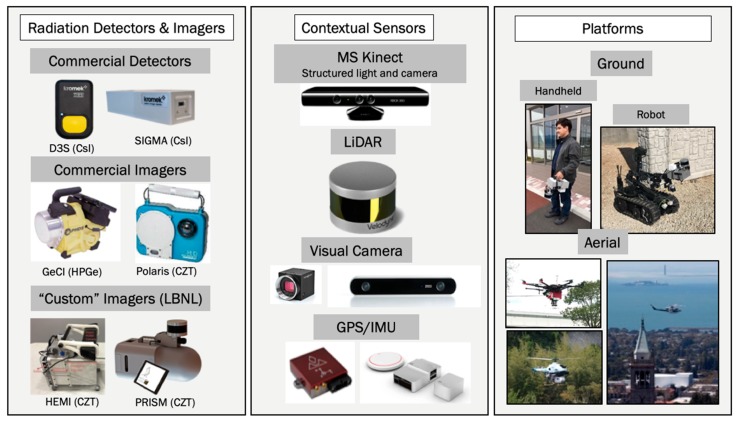
Examples of hardware components and platforms that can be used with the 3-D scene-data fusion (SDF) concept. **Left**: Commercial and custom-made radiation detection and imaging instruments can be integrated, depending on the specific requirements of the operations. These can be gamma-ray detection and imaging systems, as shown here, with neutron detectors or with combined systems, such as the recently developed Elpasolite scintillation detectors [39]. **Middle**: Contextual sensors based on structured light, light detection and ranging (LiDAR), or visual imagery can be used to map 3-D environments and to estimate the position and orientation of the instrument in this environment. **Right**: The combined radiation and contextual sensor system can be deployed on manned and unmanned ground and aerial platforms.

Figure 2 shows the hardware components that have been used for the development and demonstration of SDF for measurements in Japan, Ukraine, and across the U.S. SDF can be employed in combination with commercial radiation detectors and radiation imagers, such as the Germanium Compton Imager GeCI from PHDs [21], from H3D [22], or in combination with custom-made gamma-ray imagers, such as the High-Efficiency Multimode Imager HEMI [35,36] and the Portable Radiation Imaging, Spectroscopy, and Mapping instrument PRISM [37]. All these gamma-ray imagers represent a new generation of hand-portable instruments that combine coded aperture with Compton imaging modalities. As an example, PRISM consists of about 120 detector elements, each element serving as a detector pixel that are arranged on a spherical surface. They are arranged in a way to provide unique coding for low-energy gamma-rays for all directions. This provides omni-directional imaging capabilities for gamma-ray energies for energies below 300 keV using only active detector elements and not requiring any passive collimator or aperture mask. Not requiring the passive mask makes this instrument much lighter when compared with conventional gamma-ray imagers that are implemented with a passive mask, which is typically built of a tungsten alloy. For energies above 300 keV, the detector elements are used in Compton imaging mode, providing omni-directional imaging capabilities of up to several MeV. In PRISM, each detector element consists of a 1 × 1 × 1 cm^3^ CdZnTe detector that is implemented in so-called co-planar grid configuration [38]. This configuration improves the achievable energy resolution to about 2% at 662 keV and allowed us to determine the depth-of-interaction of gamma-rays which enhances the imaging performance. It is interesting to note that SDF does not require a radiation imaging instrument. It can be realized with simple and non-directional instruments as well, with the measured counts being projected into the 3-D scene according to the positions of the instrument which is being moved freely through the scene. 

The contextual sensing and mapping of the scene can be performed with a variety of sensors as well, including the MS-Kinect, LiDAR range finding instruments, or visual cameras. These sensors can be complemented with GPS/IMU to support the mapping and tracking of the instrument. 

SDF is characterized by its versatility and flexibility with regards to the ability to integrate with various combinations of radiation detection or imaging instrument, contextual sensor, and deployment platform. As illustrated in Figure 2, SDF-enabled systems can be operated in a hand-portable format, on unmanned ground vehicles (UGVs), unmanned aerial systems (UASs), or on manned cars, trucks, and helicopters. In this way, SDF can be widely applied for scenarios ranging from search of nuclear materials to consequence management and decommissioning. While the former is generally associated with weakly and potentially shielded sources where radiation backgrounds impact the detection capabilities, the latter is generally associated with very high and complex 3-D radiation fields where background radiation is irrelevant. It is important to note that SDF can be deployed for gamma-ray detection and mapping as well as for neutron detection and mapping. 

In summary, 3-D scene-data fusion is a powerful concept that allows for the mapping of scenes in 3-D with a freely moving system and to simultaneously fuse gamma-ray emission intensities into this scene. It enables the visualization and contextualization of radionuclide-specific radiation in 3-D and in near real-time with a system that automatically tracks its location and the measurement. Since the information is available almost immediately, the measurement can be adjusted, for example, by modifying the path or speed of the measurement. How the optimization of the measurement is realized will strongly depend on the objective of the measurement and the observation in itself. 

## 3. Results

The discussion so far has introduced the concept of 3-D SDF based on measurements employing a gamma-ray imager and the MS-Kinect. We will in the following section show more relevant examples from measurements around the world performed with radiation detection and imaging instruments in combination with other contextual sensors, particularly LiDAR. While we initially developed the concept with MS-Kinect because of its low cost, capabilities, and available open software to access the data from the structured light and visual camera, all measurements we have performed since 2015 were either with LiDAR or with visual camera-based photogrammetry. These modalities overcome the limitations of the MS-Kinect, which restricts the measurements to indoor environments and has a range of only 1–6 m.

**Figure 3:**
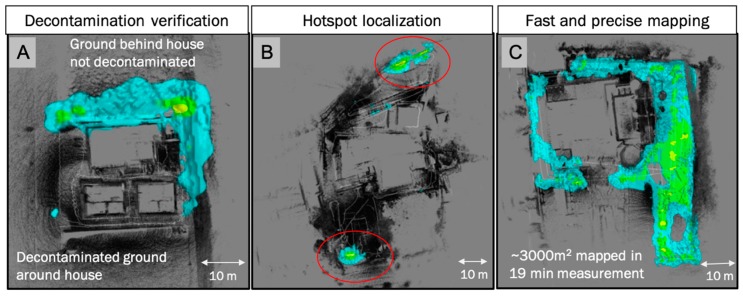
Top-down views of 3-D point clouds (black) and radiation maps (color) in evacuated urban areas in the Fukushima Prefecture. All measurements were performed with the portable gamma-ray imager HEMI in combination with a LiDAR sensor. The measurement times in these areas were always less than 20 min. (**A**) Buildings with decontaminated (1–4 μSv/hr) and not-decontaminated areas (<0.4 μSv/hr); (**B**) Building complex and parking lots with hot spots of about 10 μSv/hr; (**C**) Contaminated areas around building complex 1–4 μSv/hr).

Figure 3 shows results of measurements from locations in the exclusion zone within Fukushima Prefecture to illustrate the utilization of SDF to map radiological contamination over larger areas. All the measurements were performed with HEMI in combination with a LiDAR sensor in hand-portable configuration while walking around buildings and parking lots. The figure on the left (A) includes the decontaminated area in front of a house composed of three buildings with dose-rate levels <0.4 μSv/hr and the area behind these buildings which was not decontaminated with radiation levels of up to 4 μSv/hr. μSv/hr is a unit of dose rate as a measure of potential biological effects due to ionizing radiation, such as gamma-rays created in the radioactive decay of atomic nuclei. All materials on earth and in our universe contain some amount of radioactivity or radioactive materials (so-called naturally occurring radioactive materials or NORM). Most NORM sources are due to the radioactive decays of very long-lived radio-isotopes of U-238, Th-232, or K-40. As a reference, in the U.S., the average dose-rate due to NORM is about 0.34 μSv/hr or 3 mSv/yr. In this figure (A), the measurement paths was about 100 m and took about 5 minutes. The middle figure (B) shows the ability to effectively detect and identify hot spots as low as ~1 μSv/hr in a large area encompassing the front and rear parking lots around a building which had been previously decontaminated. The figure on the right side (C) also illustrates the ability to map contamination quickly over large areas. 

**Figure 4:**
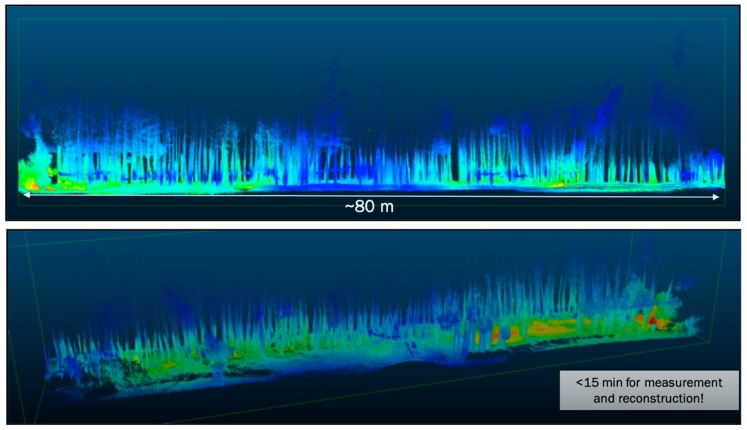
(**Top**) Fused nuclear scene maps in a contaminated bamboo forest in Fukushima Prefecture. The bamboo trees with a 10–20 cm diameter as well as bushes and ground structures are clearly discernible. Contamination was identified to be Cs-137 and Cs-134 in some areas on the ground. The original full map includes buildings located on one side of the forest but was removed to enhance the visibility of the contamination in the forest. The measurement took less than 15 minutes walking back and forth through the forest. (**Bottom**) Isomeric view of the same 3-D map as above.

While the mapping and visualization of contamination in homes and buildings, or more broadly in urban and residential areas, is critical to inform decontamination and verification activities before residents return home, it is also important to map radioactivity in environments adjacent to urban areas that are accessible to humans and animals and therefore of risk to increased radiation exposure or cross-contamination. For example, it is important to assess forested and other undeveloped areas to estimate potential contamination from these areas migrating into urban and residential areas and potentially re-contaminating them. Figure 4 shows an example of mapping contamination in a bamboo forest, also located within the exclusion zone in Fukushima Prefecture. The mapped area represents a slice of about 80 × 20 m^2^ and shows the reconstructed trees and ground with the Cs-137 contamination localized predominantly on the ground. It shows a contiguous contamination with higher contamination in some areas, specifically towards one end and in depressions. It is important to note that the advantage of using a 32-beam LiDAR sensor is the creation of dense point clouds, resulting in the mapping and registration of all trees in this forest. The whole measurement at walking speed took less than 15 min and required two paths. 

**Figure 5:**
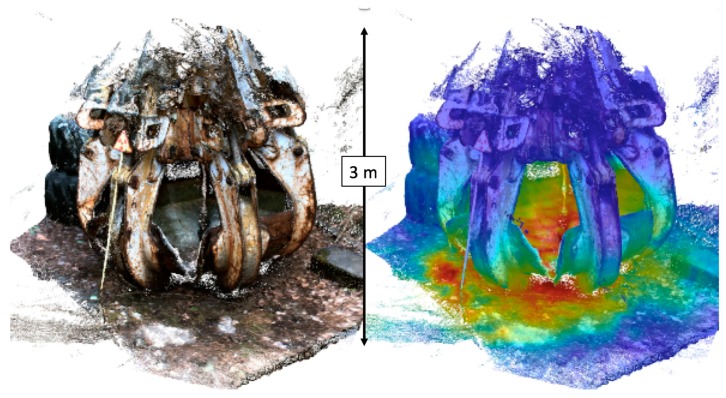
Model of the crane claw located close to the Chernobyl Nuclear Power Plant (ChNPP) in Ukraine. The left model was reconstructed via photogrammetry using visual camera data and served as the basis for the right model, which shows a fused model with the reconstructed gamma-ray emission data provided by a commercially available and hand-portable instrument integrated with SDF. The image was created for 662 keV associated with Cs-137. One can easily see that most of the Cs-137 radioactivity is inside and underneath the claw. Behind the claw, two stacked truck tires are clearly visible.

An additional advantage in this specific measurement and in the creation of 3-D digital maps is the ability to remove objects which would limit the visualization of specific areas of interest. For example, buildings on one side of the forest were removed digitally to provide an unobstructed view of the forest. Figure 3 and Figure 4 were produced using a mobile and hand-portable gamma-ray imaging system in combination with a LiDAR sensor. Figure 5 shows an example where a portable gamma-ray imaging system was fused with a 3-D surface created by photogrammetry—or more specifically stereo-photogrammetry—utilizing the visual camera on board of the SDF system. While photography is inherently a 2-D imaging modality, utilizing many different angles or projections of an object enables the creation of a 3-D digital model of this object. The object shown in this figure is a large crane claw located close to the Chernobyl Nuclear Power Plant (ChNPP) in the Exclusion Zone in Ukraine, where a major nuclear accident happened on 26 April 1986. This claw was used to remove the fuel debris from the reactor unit 4 shortly after the accident. The dose-rate levels inside and underneath of the claw are still in the order of hundreds of μSv/hr, mainly driven by Cs-137. The left-hand side of the figure shows the reconstructed 3-D model of this claw, including the radiation sign and truck tires behind the claw. The right-hand side shows the fused image based on the Cs-137 gamma-ray reconstruction at 662 keV. Most of the Cs-137 radioactivity can be seen inside and underneath the claw, consistent with separate dose-rate measurements. These 3-D fused images demonstrate the ability to detect and map contamination on 3-D objects which could be utilized for contamination mapping and monitoring on other objects in Chernobyl, in Fukushima, or elsewhere.

All the measurements discussed so far were performed with gamma-ray imaging instruments, such as HEMI and Polaris, in a hand-portable configuration. All the 3-D fused scenes were obtained by walking the instrument through the environments of interest. However, in many missions and environments, human operation should be avoided to prevent hazardous levels of exposure to radiation or other risks. In addition, in many circumstances, radiological search and mapping needs to be conducted in physically inaccessible areas. Deploying SDF-integrated systems on unmanned platforms will enable safe and effective operation in such areas.

**Figure 6:**
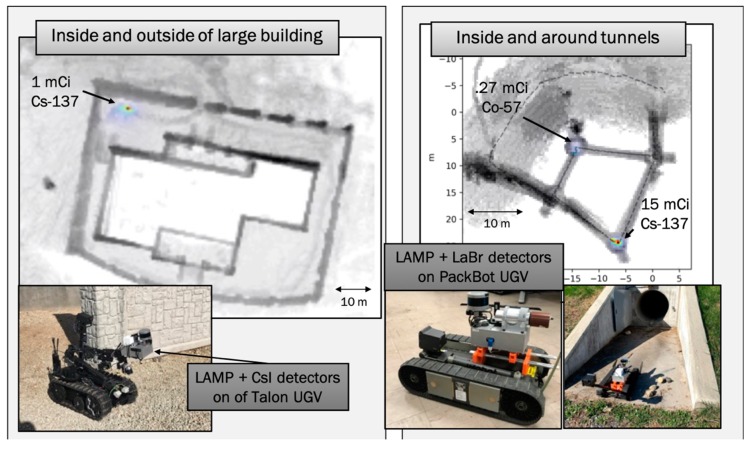
Demonstrations of 3-D SDF deployed on unmanned ground vehicles (UGVs). The localization and mapping platform (LAMP) system was combined with a 2 × 2 array of CsI detectors mounted on a Talon UGV (**left**) and a LaBr detector on a PackBot (**right**). In both cases, the non-imaging implementations of the radiation detectors were used to search for and localize sources within 3-D scenes, in or around buildings and structures (**left**) or inside tunnels (**right**).

**Figure 7:**
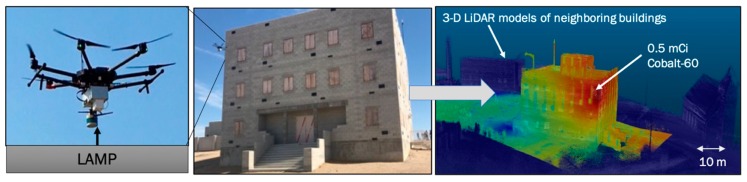
The localization and mapping platform (LAMP) in combination with a 2 × 2 CsI gamma-ray detector array mounted on a DJI Matrice-600 while flying around a building. The data product on the right shows the reconstructed model of the building (and other buildings) and the reconstructed radiation field fused with the surface of the building, pointing to the location of the Co-60 source in the correct corner of the building. The blurred image reflects the overall radiation field which was dominated by the scattered radiation in the concrete wall and the limited spatial resolution of the instrument which localized based on proximity and not based on properly imaging.

We conclude this section of examples for 3-D SDF with two specific implementations on a ground robot and on a UAS, highlighting the versality of this concept and the ability to map 3-D radiation fields fused with 3-D scenes in real-time on remotely operated, unmanned platforms. The left-hand side of Figure 6 shows the localization and mapping platform (LAMP) equipped with 4 CsI gamma-ray detectors and mounted on a Talon IV UGV. LAMP is a compact, self-sufficient package that contains a single-board computer, batteries, data storage, power control, and interfaces, that can be connected to internal and external sensors, including various configurations of radiation detectors and contextual sensors. It provides an autonomous and platform-independent realization of 3-D SDF. As shown in Figure 6 and Figure 7, LAMP with the attached detectors and sensors can easily be mounted and operated on various unmanned platforms. Mapping, tracking, image reconstruction, and data fusion calculations are performed on the computer in LAMP, and only data products are transferred via WiFi or other means of wireless communications to the user in near real-time. The left-hand side of Figure 6 shows a top-down view of the reconstructed outline of a building with a detected and localized Cs-137 point source. The source location was reconstructed to within 10 cm. The right-hand side shows the LAMP system equipped with the same contextual sensors, e.g., LiDAR, visual camera, and GPS/IMU but equipped with a single LaBr gamma-ray detector. The LaBr allows operations and gamma-ray energy measurements, i.e., gamma-ray spectroscopy, at rates of >10^6^ cps, which can be associated with a dose rate of tens of mSv/hr on the detector. The specific measurement shown was a deployment in a tunnel with the objective to find and localize radiological sources within the tunnel. The Co-57 and Cs-137 sources were accurately detected and localized inside the reconstructed 3-D model of the tunnel. The localization accuracy was about 15 cm. 

The final example shown in Figure 7 illustrates the operation of LAMP with 4 CsI gamma-ray detectors on board on a UAS, specifically the Matrice-600 manufactured by DJI [40]. The human-controlled flight around the three-story building took about 13 min and resulted in the reconstruction of all buildings in the vicinity of up to 100 m consistent with the range of the LiDAR and the accurate detection and localization of a Co-60 source in the corner room of the third floor in one of the buildings. Other means of ground-based deployments would have taken significantly longer than the 13 min flight time and only SDF enables the integration of the radiation field and the location of the source in 3-D within the building. Similar to the deployment of LAMP on the UGV, the deployment of the UAS was done with non-imaging radiation detectors, still resulting in the localization of the sources in 3-D. This was enabled through proximity imaging or localization utilizing the underlying 1/r^2^ dependency of the distance between the source and the moving detection platform creating the necessary projections for the reconstruction. While the use of gamma-ray imaging systems provides higher resolution and sensitivity in the localization of radioactive sources, even non-imaging instruments can be used to localize compact sources with SDF based on the enabled motion of the instrument. 

## 4. Discussion

Scene-data fusion is a concept that is based on the integration of computer vision with nuclear radiation detection and imaging technologies, providing new means to map and visualize nuclear radiation in 3-D. It enhances the accuracy, speed, and effectiveness in many applications related to the detection and mapping of nuclear and radiological materials—in the search and characterization of materials to enhance nuclear security, in the monitoring and management of nuclear legacy sites and operating nuclear facilities, in radiation protection more broadly (for example, in accelerator facilities), in consequence management in response to the intentional or unintentional releases of radioactive materials, and in the decommissioning of nuclear facilities. In all these areas, SDF will have a substantial impact not only in the effectiveness but also in the safety of the operations. This is due to the ability to deploy SDF on a multitude of remotely or autonomously operating platforms, such as drones or ground robots, and the ability to provide immediate feedback for the operations. 

The localization and mapping platform (LAMP) realizes this concept via the utilization of contextual sensors, such as LiDAR or visual cameras, that can be combined with almost any imaging and non-imaging radiation detector. We have demonstrated SDF with LAMP-based systems in contaminated areas in Fukushima and Chernobyl and in search missions in the U.S. Of specific interest is the ability to map radiological contamination and to verify decontamination activities in urban environments and in radiological and nuclear facilities. Such an assessment is critical to minimize the risks of operators and the public to inform evacuation, decontamination, and resettlement efforts. In addition, with the reconsideration of tactical nuclear weapons, military forces are concerned with enhancing available capabilities to map fall out to avoid exposure to troops and equipment [41]. 

In summary, SDF in combination with LAMP facilitates effective and smart ways to “see” sources of nuclear radiation in 3-D. While the focus to-date was in the mapping of gamma radiation, recent advances in detector materials—specifically so-called Elpasolite scintillators—provide not only gamma-ray detection but also neutron detection, localization, and mapping capabilities. Neutrons provide complementary information about so-called special nuclear materials such as Pu-239 that emit gamma-rays and neutrons. In contrast to gamma-rays, neutrons cannot be shielded with shielding materials such as lead. Therefore, the extension of SDF to detect, map, and visualize neutrons will enhance the ability to search for generic radiological as well as special nuclear materials. An additional enhancement of SDF will be in the operation of several platforms to create larger-scale 3-D maps. Overall, SDF enables radionuclide-specific gamma-ray vision in 3-D that can be extended to other types of radiation such as neutrons or even beta particles in the future.
